# Health and health literacy from the child’s perspective: a qualitative study in 9–12-year olds

**DOI:** 10.1093/heapro/daae208

**Published:** 2025-02-11

**Authors:** Wieke Van Boxtel, Mai Chinapaw, Katarina Jerković-Ćosić

**Affiliations:** Research Group Innovation in Preventive Healthcare, HU University of Applied Sciences, Heidelberglaan 7, 3584 CS Utrecht, The Netherlands; Public and Occupational Health, Amsterdam UMC location Vrije Universiteit Amsterdam, De Boelelaan 1117, Amsterdam, The Netherlands; Health Behaviors and Chronic Diseases, Amsterdam Public Health, Amsterdam, The Netherlands; Public and Occupational Health, Amsterdam UMC location Vrije Universiteit Amsterdam, De Boelelaan 1117, Amsterdam, The Netherlands; Health Behaviors and Chronic Diseases, Amsterdam Public Health, Amsterdam, The Netherlands; Research Group Innovation in Preventive Healthcare, HU University of Applied Sciences, Heidelberglaan 7, 3584 CS Utrecht, The Netherlands

**Keywords:** health literacy, children, health perception, qualitative methods, focus groups

## Abstract

To promote health literacy (HL) in children, it is crucial to recognize their unique social environment, experiences, and comprehension of health and HL. This study aims to gain insight into the perspectives of children aged 9–12 years regarding health and HL within the context of health promotion. We conducted focus group discussions with children at three primary schools in the Netherlands, covering (i) What health means to children (ii) How they engage with health information (HL), and (iii) How they respond to the items of an HL questionnaire (HLS-Child-Q15-NL). Thematic analysis was used to analyze data from nine focus groups with 46 children. We identified three themes: (i) perspectives of health and healthy living, (ii) information for healthy living, and (iii) sources of information for healthy living. Children rated being yourself, physical activity, and happiness as the most important health topics. They receive health information passively, mainly from caregivers and social media, and emphasize the need for simple language. Trustworthiness of sources and usefulness of information were also important. Responses to the HLS-Child-Q15-NL varied, with some items being unclear and leading to misunderstandings, though generally seen as easy. The findings highlight children’s broad perspective of health and HL. Health-promoting activities should focus on developing HL skills related to children’s lived experiences and health topics that they deem important. This will present an opportunity for children to engage with health information more actively and critically.

**Contribution to Health Promotion**

- Children view health broadly, valuing being themselves, staying active, and being happy.
- Health-promoting activities should focus on daily life skills and important health topics for the children involved.
- Encouraging active and critical engagement with health information helps children make better health choices.

## BACKGROUND

Living a healthy lifestyle can be challenging in today’s society, as children are exposed to a multitude of sources of health information that vary in quality. To effectively utilize this information, children need to develop health literacy (HL) skills among other life skills. From a health promotion perspective, HL encompasses a wide range of health-related activities and the quest for information in everyday life, such as media influences and digital technologies (Velardo and Drummond 2017). When children have the “knowledge, motivation and competence to find, understand, appraise and apply health information” appropriately, they are more likely to develop and maintain healthy lifestyles (Sørensen and Okan 2020).

Promoting children’s health requires appropriate health information, including easily accessible and understandable information for all age groups (Velardo and Drummond 2017; Okan et al. 2019). Bröder et al. (2019b) argue that from a very young age, children gain experiences, form opinions, and develop their unique understanding and view of the world around them, including their own health and well-being. Velardo and Drummond (2017) suggest that addressing children’s specific HL needs can have a lasting impact on the formation of attitudes and behaviors that persist into adulthood. In addition, it promotes their active involvement in their health for the development of self-efficacy, ownership, and empowerment (Simovska and Jensen 2009). Bröder et al. (2019a) suggest that the lack of HL and health-promoting skills in young populations could lead to an increased risk of adverse health outcomes and increased healthcare costs. HL skills and health-promoting abilities change at different stages of child development. For example, independence increases during adolescence and adolescents need more autonomous decision-making skills (Velardo and Drummond 2017). Consequently, these changes deserve attention when studying HL during the transitional stages of child development.

Recent studies (Deyra *et al.* 2020; de Bot and Dierx 2024) indicate that children have a broad and diverse understanding of health, including all six dimensions of *My Positive Health* (MPH): my body, daily life, my feelings and thoughts, now and the future, feeling good about yourself and participation (Huber *et al.*, 2016; Institute of Positive Health, n.d.). They identify social and interpersonal determinants of health. Children also tend to have positive attitudes toward health promotion and knowledge of health-related topics (Deyra *et al.* 2020). Despite these findings, evidence remains scarce on how children construct their understanding of health-related concepts in their daily lives (Fairbrother et al. 2016; Bröder et al. 2017; Okan et al. 2019). Specifically, there is a notable gap in research and theory regarding how children engage with health-related information and whether they find information meaningful, as previously noted by Fairbrother et al. (2016).

Currently, children’s perspectives are mostly outlined by adults without involving children and adolescents in research on HL skills, knowledge, and health behaviors that are important to them (Bond and Rawlings 2019). To gain a holistic understanding of children’s health, Velardo and Drummond (2017) argue that it is essential to include the child’s perspective alongside the valuable work being done with caregivers.

To contribute to developing meaningful initiatives for developing HL and promoting health in children, we need to recognize and respect the child’s distinct social environment, experiences, viewpoints, and comprehension of health and HL (Velardo and Drummond 2017). Therefore, to develop optimal health-promoting activities this study aims to gain insight into the child’s perspectives of health and HL.

## METHODS

We conducted a qualitative study using focus group discussions and reported in accordance with the Consolidated criteria for reporting qualitative research (COREQ; Tong et al. 2007) (Supplementary File 1). This study was approved by the Medical Ethics Committee of Amsterdam UMC under number: 2022-0036.

### Sample

The convenience sample consisted of three primary schools in two different cities in the Netherlands. Because of the exploratory nature of our study, we have not selected participants based on gender or other demographic characteristics. The socioeconomic status of the neighborhoods these schools are located in is slightly higher compared to the Dutch mean, meaning that parents of the children included in the study might have middle or high socioeconomic positions (CBS 2023; DUO 2024). The schools had differing pedagogical approaches and no dedicated health education curriculum in which health or HL was taught. After the agreement of the principals and teachers, children aged 9–12 years (Dutch grades 6, 7, and 8) and their caregivers received an invitation to participate by email or message in the school app. In addition, before participating in the focus group discussions the children took home an information letter with a consent form on paper to be signed by their caregivers.

### Instruments

A semistructured focus group discussion protocol was developed by researchers R.H. and W.v.B., which encompasses the developmental level of the children in education and pedagogy (expertise of RH) and in health and HL (expertise of W.v.B.; Supplementary File). We planned to discuss three topics: (i) what health means to children, what their perspectives on the domains of MPH (Institute of Positive Health, n.d.), (ii) how they engage with health information (HL), and (iii) how they respond to the items on the HLS-Child-Q15-NL questionnaire on HL (Hahnraths et al. 2021). The first topic started with an open question, that is, what do you think about when you think about health? The children first wrote down their answers individually and then shared them in the group. A topic list was made for discussing topics 2 and 3 to keep the children focused on the topic, and also when taking into account the concentration span of the participants, to create space for more information and details by using open-ended and closed questions (Delfos 2020). We chose the HLS-Child-Q15-NL questionnaire because this is the only questionnaire for HL in children in Dutch and it is commonly used in Europe.

An iterative process was used to adapt the topic list and protocol to what the children needed during the focus group discussions. For example, after the first two focus groups, we noticed that children found it difficult to think of health topics without first being provided with some examples. Therefore, we decided to bring a print of the MPH spider web for children (Institute of Positive Health, n.d.) and place it on the table as a discussion starter (Supplementary File). This conversation tool allowed children to come up with more examples which broadened the discussion. In the last two focus group sessions, we started with the open question and introduced the spider web when needed. As a final assignment in all focus group discussions, we used the spider web to ask the children what they rated as the most important health topics.

### Process focus group discussions

The focus group discussions were conducted in a private room at each school and lasted 30–45 minutes. At schools A and B (April 2022), the discussions were moderated by R.H., and at school C (July 2023), by W.v.B. in order to share and observe the diversity of perspectives (Adler et al. 2019). No teachers or school staff were present during the focus group discussions.

### Data preparation and analyses

The focus groups were audio-recorded and transcribed verbatim in Dutch using Amberscript and MS Word. Transcripts were checked using the audio by a researcher who was not present at the discussions. No member checks of the transcriptions or outcomes were performed due to the children’s difficulty in reading and understanding the transcripts.

The thematic analysis of Braun and Clark (2006, 2019) was used to identify overarching themes inductively and to provide a rich and detailed account of the children’s perspectives on health and HL (Vaismoradi et al. 2013). The first steps of the analysis were conducted by two researchers (R.H. and W.v.B.) who facilitated the focus group discussions. They first familiarized themselves with the data and then independently performed open coding of the segments. Codes were discussed and clustered based on similarities and consensus. Saturation was reached when no new codes were needed in the data. The clustered codes were then searched for aspects of health and HL. Subsequently, we reviewed the aspects based on the data from all three schools, which showed a good fit with the concepts of HL and MPH, making the data theoretically sound. To give insight into the children’s perspectives, we generated overarching themes based on the content discussed by the children. W.v.B. and K.J. performed the following steps of the analysis. W.v.B. reviewed the coded segments again and proposed overarching subthemes. K.J. and W.v.B. then discussed the subthemes and underlying codes, identifying overarching themes in which all subthemes were clustered. The theme names were then agreed upon by consensus. Vivid and compelling quotes were selected from the analyses to illustrate the results. Saturation would be reached if no new codes emerged from the data. The analysis process was performed in ATLAS.ti 22. Themes, codes, and quotes were translated into English after completing the analyses.

## RESULTS

We invited approximately 200 children and received 30 consent forms from School A, 9 from School B, and 12 from School C. A total of nine focus group discussions were held with a total of *N* = 46 children (Table 1). Five children who had provided consent were not at school when the focus group discussions were organized and therefore did not provide data.

**Table 1.** Participant characteristics (*N*, age, gender and grades) per school and focus group.

	N	Age 9	Age 10	Age 11	Age 12	Gender		Grade 6^b^	Grade 7	Grade 8
						Boy	Girl			
School A	26	5	5	7	8	10	16	9	3	14
FG^a^ 1	5	2	2	1		3	2	5		
FG 2	4	3	1			1	3	4		
FG 3	3		2	1		1	2		3	
FG 4	7			5	2	4	3			7
FG 5	7			1	6	1	6			7
School B	8	1	5	2		4	4	4	2	2
FG 6	4	1	3			1	3	4		
FG 7	4		2	2		3	1		2	2
School C	12	1	4	6	1	8	4	4	7	1
FG 8	6		1	5		4	2		6	
FG 9	6	1	3	1	1	4	2	4	1	1

^a^FG—Focus Group, Grades are last three grades in Dutch primary schools.

^b^Dutch grades 6, 7 and 8 are last three grades in primary school.

### Themes

**Figure 1.**
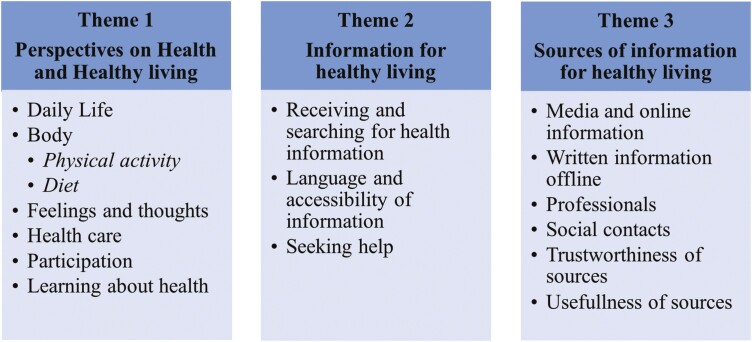
Identified themes and subthemes: Theme 1: Perspectives on health and healthy living; Theme 2: Information for healthy living; and Theme 3: Sources of information for healthy living (code tree and networks for these themes are presented in the Supplementary File).

We identified three themes (Fig. 1). Theme 1 emerged from the open question of what children think about when thinking about health and what their perspectives on the domains of MPH (Institute of Positive Health, n.d.). Theme 2 describes the health information topics that children search for and receive as well as what they feel is accessible in terms of language and form, and how they seek help understanding and using the information. Theme 3 describes the sources of health information that children use, their understanding of them, and how they appraise the trustworthiness and usefulness of the sources. HL aspects such as finding, understanding, appraising, and applying information are described in themes 2 and 3.

### Theme 1—Perspectives on health and healthy living

The topics that the children considered relevant to their health are presented on the left-hand side of Table 2. The primary topics children discussed were diet, physical activity, and being outside. The children from schools A and B only mentioned topics related to their body such as diet, physical activity, sleep, or illness in their session. Consequently, we introduced the MPH spider web to inspire them to think about what health could mean beyond their body. In contrast, the children from School C introduced more diverse health topics. They demonstrated a broader perspective on health, with a focus on well-being, by mentioning topics such as relaxation and emotions. As a final assignment, all children were given the MPH spider web to indicate what they felt were the most important health topics. The top 10 of the most important health topics are presented on the right-hand side of Table 2. The children emphasized that activities of daily life and participation including being yourself, being happy, going to school, and having friends were more important than aspects of their body which include a healthy diet and physical activity.

**Table 2.** Health topics children mentioned (left) and rated as most important (right) by children.

Health topics mentioned by children	Top 10: Health topics children rated as most important
Healthy diet	Being yourself
Physical activity	Sports and exercise
Being outside	Being happy
Emotions	Healthy diet
Illness	Going to school
Sports	Sleep
Relaxing	Friends
Gaming	Leisure time
No substance use	Being cheerful
Sleep	Having energy
Hygiene	
Medication	
Being treated well	
Environment	
School	
Being fit	
Making your own choices	
Talking	
Work	

When discussing **diet**, the children mostly mentioned fruit and vegetables as being healthy. However, some children discussed the need for protein and vitamins. They discussed foods such as sweets and chocolate, indicating that these are not healthy, some mentioned unhealthy drinks, not all children knew that fruit drinks contain a lot of sugar. Questions from the HLS-Child-Q15-NL regarding diet were mostly understood correctly.

*“Iron makes you strong. And that keeps you in good health and that and the vitamin CD. C is fruit actually, eat enough fruit and B and D. I don’t really know exactly.” (10-year-old girl, FG 8).*

Regarding **physical activity and sports,** the children mentioned it is important to be physically active and play sports. A couple of children suggested more physical activity in school during lessons and indicated that it would also be nice to be outside more during a school day.

**“*We very often want to have outdoor lessons, but it’s not allowed. [...] Yes, more exercise because sometimes you sit still for a few hours and then you get back pain.” (10-year-old boy and girl, FG 3).*

Regarding **daily life**, children discussed the importance of having time off to do things with friends, to relax, and to be outside. When they were thinking about health, many children mentioned playing outside. Some related this to having energy, and to releasing too much energy.

*“If you’re just active, you need to get rid of your energy” (11-year-old girl, FG 9). “And that’s healthy, too. Getting rid of your energy.” (9-year-old girl, FG 9).*

Questions from the HLS-Child-Q15-NL regarding daily life were mostly understood correctly, on traffic rules for example. However, when the question is not specific on a topic, for example, ‘How easy or difficult is it to understand what your parents tell you about health?’ or ‘How easy or difficult is it to judge what helps a lot for you to stay healthy and what does not help much’ children tend to reformulate the question into their answer or do not answer based on experience.

Feelings were discussed in terms of emotions. Being happy as well as being cheerful is in the top 10 most important health topics. On the other hand, children indicated it is important to have feelings and emotions, and that showing these feelings is healthy.

*“Well to me it’s important. Suppose you never cry, suppose you are never angry, suppose you are never happy, then actually you are never anything. Actually, then you are completely stuck and then you aren’t healthy.” (11-year-old girl, FG 8).*

Some children shared experiences they had with illness or **healthcare** but also indicated they do not know much about it. A few children mentioned pain relief medication and discussed vaccinations. Additionally, some of their caregivers were health professionals from whom the children learned more about health. Questions from HLS-Child-Q15-NL regarding healthcare appeared to be a bit more difficult to answer because not all children had experience of healthcare.

**Participation** included having friends and being able to do things together. The children discussed the formulation of items such as bullying; they suggested that preventing bullying should be considered healthy. Moreover, they indicated that the inclusion of negatively formulated items in a health framework impacted their perceptions.

When asking children what they want to **learn about health**, younger children (nine-year-olds) indicated that they *“don’t necessarily need to know anything. […] Because I don’t feel I have the need to.”* Eleven- and 12-year-olds indicated they did not know all specifics of a healthy diet and stated there are *“Things we’ve never heard about.”.* A few children said that they did not know what was healthy and what was not, both in general and concerning food. On the contrary, a few children talked about their knowledge of having energy and their daily lives. Some talked about having health professionals in their family and therefore talking more about health at home. Illness and feeling limited or disabled were touched upon but not discussed by the children.

### Theme 2—Information for healthy living

This theme includes how children receive health information, what the language and form of this information should be, and when they seek help regarding health information.

#### Receiving and searching for health information

Children receive a lot of health information without searching for it. In their daily lives, they get information from their caregivers, school, and also through social media.

At home, most children learn implicitly and explicitly about health. They stated that they get health information from their caregivers and family members. This information is about personal hygiene, sleep, physical activity, and a healthy diet. Some children get information about health risks such as smoking and alcohol consumption at home. Most children do not have to actively ask or search for health information. One child indicated that it should be part of his upbringing and the responsibility of the caregivers.

*“I don’t really learn about health. My caregivers take care of that.” (10-year-old boy, FG 9).*

When children actively look for health information, they ask caregivers or family members about it most of the time. Usually, their questions are answered, but if not, some caregivers look for the answers on the internet.

*“Or if I ask my caregivers and they don’t know, they look it up on Google.” (11-year-old girl, FG 5).*

The children indicated that they learn about health in school. However, some children doubted or could not answer if or how school pays attention to health. Children mentioned some aspects of health that they wanted to learn about in school, for example being outdoors more or having more lessons outdoors. Other children discussed how to get along with people through school. Regarding a healthy diet, some children indicated that school does have rules on this, but these are not always complied with.

*“My mom makes me bring fruit to school every day, and everyone else brings cookies.” (11-year-old girl FG 4).*

Searching for health information and asking questions about health was seen as a school task, not necessarily something children usually do by searching for information themselves. Some children stated they do not actively search for health information. Moreover, they did not know what to search for. This does not mean children do not search for any information, they search for topics other than health.

*“In class, we had to look something up on the internet and then you get all these websites that are useless.” (10-year-old girl, FG 3).*

#### Language and accessibility of information

Children discussed how information should be provided and what sort of language should be used. They talked about what information they understand and how to find understandable information. The language that children indicated as understandable comprises simple words, with important words printed in bold, and short texts.

*“I often check to see if the correct words are used, that they explain it clearly and then I look a little further to see if it is child-friendly.” (11-year-old boy, FG 7).*

The children indicated that they do understand information about health in terms of explanations of medical reasons. Information or explanations given in school are also usually understood. They talked about online information being understandable if it is related to their own experiences. Online information was often—but not always—understood. The children indicated that it is important to formulate a good search query to find understandable answers. Sometimes they have to ask for extra explanations or read other information.

In the conversations, the children talked about why they do not understand information. If the information was not useful, too extensive, contained difficult words, or if the children did not listen, they misunderstood the information. When information was given in conversation and people talked too fast, the children also found it more difficult to understand. Some children would rather watch an information video than read it somewhere.

#### Seeking help

If the information was not understood the children try to seek help. They ask caregivers, and teachers or search online for answers or explanations. However, one child also indicated that caregivers do not know everything. Some of the children pretended they understood something and did not ask for, or search for, an explanation.

*“I don’t really have an example, it’s just that sometimes I just don’t understand. it’s annoying [...] Then I ask again but then they say the same thing. Some things are super hard to understand.” (9-year-old girl, FG 2).*

### Theme 3—Sources of information for healthy living

This theme describes the sources children use to find information they are interested in, and what they think about the trustworthiness and usefulness of sources.

#### Social contacts and professionals

The primary sources the children spoke about were their caregivers, family members, teachers, and doctors. Caregivers or family members were the main sources of information. Children said they actively asked them for health information or asked for help in finding health information from another source. Moreover, some children said they only receive health information or learn about health at home.

*“Usually then I ask my mom, and if she doesn’t know, she looks it up on Google and tells me.” (11-year-old girl, FG 4)*

School teachers or sports coaches were also mentioned as sources by the children. They indicated that they could ask their physical education teacher or soccer coach questions about sports or physical activity.

#### Media and written online and offline information

The secondary sources children use for finding health information are search engines such as Google, YouTube and TikTok, videos, TV programs, Wikipedia, books, packaging/labels, or school programs.

*“[…] actually, health [information] can be found anywhere, but sometimes you have to look for it.” (12-year-old boy, FG 9).*

Children indicated that they do come across health information on YouTube and TikTok. They often saw health-related videos or information on how to prepare food or do exercises on social media. Once they have engaged with health-related videos, they receive related content on further occasions. Some children search for health information on YouTube or TikTok if they cannot find an answer from a search engine. They mentioned TikTok more often than other social media platforms. Some children also came across videos or TV programs (such as educational or news programs for children) in which health was a topic. However, they used these sources less than online sources, social media, or streaming services such as Netflix or Disney+.

*“On TikTok, you just get a video, like they explain something and then it’s filmed, and on Google, you have to read it and I don’t like that.” (12-year-old girl, FG 5).*

Books were mentioned as offline secondary sources in answers to a direct question about where to look for information other than online, or when books were proposed as an option. Food or packaging labels were brought up in their answers to the question about how difficult it is to find out what food is healthy. A child mentioned the risk warning on cigarette packages when he discussed how he gave his mother health advice about smoking.

#### Trustworthiness of information

The trustworthiness of health information was discussed in terms of reliable sources, and when they thought a source would be reliable. They also talked about trust and truth, which is difficult to appraise. The children understood the importance of a reliable source, they indicated multiple sources to be more reliable than the internet, and they thought that the information must be true. Moreover, if they were not sure about the trustworthiness of sources or information they asked their caregivers. Regarding the reliability of the internet, the children talked about information being reliable if the majority gave the same answer. Some children thought the first results from their search were the most reliable. On the other hand, the children knew that quite a lot of information was fake or edited, which made it difficult to appraise whether the information was correct or real. Some children had lessons in school on appraising information, for example, to detect fake news. Wikipedia was mentioned in two focus groups as one of the more reliable sources of information.

#### Usefulness of information

Information was found useful if it provided an answer to their question. However, they did not always get the answers they searched for. Furthermore, the children indicated that using the right information sources, comparing the sources, and then applying the best result is important to find useful health information.

*“[…] sometimes Google doesn’t understand my question and then I get completely different information.” (9-year-old girl, FG 9).*

The children talked about applying information when this information was provided by a reliable source, such as their caregivers. Some children decide to apply information and do not need help or reminders. However, the majority of children need some reminders to do things such as drink sufficient water or brush their teeth.

Children indicated that they did not apply received or found health information if it was not perceived as beneficial. Although reliable sources provide useful information, this is not a guarantee that children will use the information. They indicated that advice from caregivers or health professionals was not always acted upon. Moreover, they forgot to follow the advice or do not feel like doing it. Children also discussed the influence of their environment on making healthy choices.

## DISCUSSION

This study aimed to delve into the perspectives of Dutch children aged 9–12 concerning health and HL. It was important to acknowledge and value social environments, personal experiences, and understanding of children regarding health and HL. This age group represents a critical developmental stage, where attitudes toward health and well-being begin to solidify, potentially influencing lifelong health behaviors.

The findings from our nine focus group discussions revealed three primary themes with insightful outcomes:

- **Perspectives on health and healthy living:** Children rated being yourself, physical activity, and being happy as the most important health topics. A healthy diet and physical activity were prominent in the discussions. In addition, notable mentions in the field of emotional well-being were being yourself and being happy, and in the field of social dimensions were health, friendship, and school life.
- **Information for healthy living:** There was a tendency toward passive engagement with health information, primarily through parental guidance and social media channels. Children indicated cases where information is easily found and understood in terms of language and form. For example, using simple wording in short texts.
- **Sources of information for healthy living:** Reliance on caregivers and social media as information sources was prominent. However, discussions also highlighted a critical approach to evaluating the trustworthiness and usefulness of health information, albeit with variations in the level of critical appraisal skills across the age groups.

Similar to the findings of de Bot and Dierx (2024), we also found a diverse and broad conceptualization of health. Children reported a broader concept of health as important when prompted with the MPH spider web than when thinking about health before being prompted. In developing the MPH tool for children, researchers found that children conceptualized health in terms of lifestyle or daily activities (de Jong-Witjes et al. 2022). We found similar results, with the children reporting things such as a healthy diet, physical activity, and being outdoors as being what they think about in terms of health. In a Swedish study of children’s perceptions of health, “associating with others” was found to be an important health issue (Kostenius 2008, p. 38) In our study, this sense of togetherness was not that clear. However, the children in our study mentioned friendship, and going to school as important health topics.

We did not ask the children to define HL, but they discussed how they find, understand, evaluate, and apply health information. Children reported that they need knowledge about health and that they search for information online or ask their caregivers. This is consistent with Joseph and Fleary’s (2021) findings that adolescents define HL as health knowledge, understanding of health terms and information, and skills for acquiring health information. Our study suggests that 9–12-year olds are also exploring and using HL skills to engage with the health information they receive. However, they reported difficulties finding, understanding, communicating, appraising, and applying health information in some circumstances. This suggests an educational opportunity for more active and critical engagement with health information among this age group.

The role of caregivers as a primary source of health information for children aligns with previous studies. Our findings, similar to those of Michaelson et al. (2021) and Fairbrother et al. (2016), indicate that children passively receive information regarding health and need help with finding and applying health information, mostly from family sources. As stated by Michaelson et al. (2021) “Throughout infancy and childhood, we live among others who can provide for our basic needs, guide and nurture us as individuals, and launch us on health trajectories that follow us throughout the life course” (p. 2). Consequently, from this perspective, it also appears logical that children are passive recipients of health information, as the responsibility of health shifts from the parent to the child during late childhood and adolescence.

### Implications

It is beneficial for children to live in health-promoting settings (Michaelson et al. 2021). In our study, the children indicated that they rely on their social contacts, family, and professionals to deliver health information. Characteristics that contribute to health promotion in families are health-promoting behaviors and habits such as a balanced diet, activity behavior, and good sleep, which the children in our study indicated as important health topics. When children learn to actively engage with health information and share it with their families, it can benefit the entire family. For instance, in engaging in health-promoting activities children can share their broad perspective on health with their families.

Children must learn to actively engage with health information and acquire the knowledge and skills to find, understand, discuss, evaluate, and apply it. In educational settings such as primary education, efforts are being made to incorporate HL into curricula. Our scoping review describes several international studies and education reports that describe learning outcomes for children to learn HL (Van Boxtel et al. 2024). The core objectives for primary education in the Netherlands (SLO 2006) do not include descriptions of HL. However, there are opportunities to integrate HL into the core competencies of primary education in the Netherlands by linking HL skills to specific objectives related to subjects such as the Dutch and English languages, mathematics, physical education, biology, and orientation to oneself and the world around you. Research on integrating HL into education is needed to develop health-promoting activities in schools. The findings of this study provide valuable insights for co-creation sessions with children to develop such school activities.

We found that children discussed the trustworthiness of information and related this to sources and online information that might be edited or fake. The digital and online sources of information used by and available to children are changing rapidly. Adults need to know and understand the online platforms from which children receive information. This is not just about the information children seek, but more importantly, the information they are prompted to see. In line with children’s responsibility, they need support and learn HL skills to deal with this information. There is a need for critical appraisal of health information, especially for open online sources such as social media, where information is attractively presented to children. Critical appraisal requires active engagement with information and is a skill children learn in most educational settings. More recently, critical appraisal as an HL skill has been linked to digital literacy in education settings. Core competencies from digital literacy are used to teach and learn about health through digital HL (Okan et al. 2020; Schulenkorf *et al.* 2021). Searching for matching core competencies within curricula and using these for health-promoting activities and developing HL in schools seems a promising way ahead.

### Strengths and limitations

A focus group provides an opportunity for interactive discussion and sharing of personal experiences. In addition, a group can encourage children to speak their minds. It also allows children to help each other if something is not understood. In the Dutch school system, children are used to sharing and participating in conversations with groups of peers of similar age. To best understand children, we used an insider’s perspective (Migchelbrink 2018). Therefore, we tried to stay as close to the children’s environment as possible. We did not conduct member checks with the children due to time constraints, and the difficulty of reading and understanding the transcripts.

Our results might be influenced by the medium to high socioeconomic positions the schools are located in. Around 15% of the invited children participated in the focus groups, which might indicate selection bias, as it is common that highly-educated caregivers tend to provide parental consent more often than caregivers with a lower level of education. However, we have no information about children’s social economic positions and family composition. Nevertheless, we tried to be inclusive by using simple language in the information letters and diverse ways of informing caregivers e.g. letters, short messages in the school app.

## CONCLUSION

This study highlights the broad perspective of health and HL discussed by the children which extends beyond physical well-being, encompassing emotional health and the impact of social environments on health. As children describe health from their personal experiences and daily life it is important to tailor health-promoting activities to these insights. We therefore recommend including the child’s perspective on the development of health promotion content, by co-creating health promotion activities with children instead of asking them to only evaluate an activity afterward. Regarding the accessibility of health information, it is especially important to use simple and comprehensible language and easily accessible information, where sentences are short, and important words are printed in bold. The involvement and co-design with the target population could also be valuable in the assessment of accessibility and development of (new) health information.

## Supplementary Material

daae208_suppl_Supplementary_Files

## Data Availability

The data underlying this article will be shared on reasonable request to the corresponding author.
