# Supplementary material for: Health and health literacy from the child’s perspective: a qualitative study in 9–12-year olds
Source: Health Promot Int. 2025 Feb 11;40(1):daae208. doi: 10.1093/heapro/daae208 (PMC11811769; doi:10.1093/heapro/daae208)

**Supplementary file - Health and health literacy from the child’s perspective; a qualitative study in 9-12-year-olds**

Van Boxtel, W., Chinapaw J.M.M, Jerkovic-Cosic, K.,

Table of Contents

[1. Semi-structured focus group discussion protocols 2](#_Toc178328820)

[Version 1 – School A and B in Dutch 2](#_Toc178328821)

[Adapted version – School C in Dutch 7](#_Toc178328822)

[2. COREQ (COnsolidated criteria for REporting Qualitative research) Checklist 11](#_Toc178328823)

[3. Code tree 13](#_Toc178328824)

[4. Code networks for themes from Atlas.ti in Dutch 18](#_Toc178328825)

[5. Code networks of HL aspects 21](#_Toc178328826)

## Semi-structured focus group discussion protocols

## Version 1 – School A and B in Dutch

Hoofdvraag:

Wat vinden kinderen in de leeftijd 9-12 jaar belangrijk als het gaat om in het inzetten van gezondheidsvaardigheden om dagelijks gezond te kunnen leven?

Benodigdheden:

- Rustige ruimte
- Opname apparatuur (telefoon)
- Voldoende A4 papier
- Pennen
- Namenlijst
- Dit protocol uitgeprint

Begin van elke opname melden hoeveel kinderen er aanwezig zijn bij het gesprek, welke klas en welke leeftijd.

| Welkom: | Ik ben Renée en ik moet voor school een onderzoek doen naar de gezondheid van kinderen. Daarbij wil ik heel graag weten wat jullie mening is en wat jullie belangrijk vinden als het gaat over gezondheid.  Ik ga straks dus wat vragen stellen over gezondheid en dan mogen jullie met elkaar in gesprek gaan en jullie eigen mening geven. Dit duurt ongeveer een halfuurtje.  Ook neem ik het gesprek op met mijn telefoon zodat ik het later terug kan luisteren, je hoort alleen je stem en verder blijf je anoniem  Hierbij zijn er wel een paar regels zodat het gesprek goed gaat. |
| --- | --- |
| Regels: | - We luisteren naar elkaar en praten niet door elkaar heen - Je mag in gesprek gaan met elkaar - Er is geen antwoord goed of slecht. - Het is belangrijk dat je je eigen mening geeft, deze hoeft dus niet hetzelfde te zijn als die van je klasgenoot. - We lachen elkaar dus niet uit en hebben respect voor elkaars mening   Zijn er verder nog vragen? |
| Start focusgroep discussie | Jullie kennen elkaar al dus we hoeven geen voorstelrondje te doen.  Iedereen heeft een papier voor zich en een pen. Daar mag je even je leeftijd op schrijven, en het antwoord op de volgende vraag: |
| Onderwerp 1:  Gezondheids-onderwerpen | - Als het gaat over gezond leven, waar moeten jullie dan aan denken?   *Je mag het opschrijven, maar als je tekenen makkelijker vindt mag dat ook. Je mag meerdere dingen opschrijven en je mag er best even over nadenken.*  *Pennen inleveren, en antwoorden met elkaar doornemen.*  Mijn positieve gezondheid web er bijhalen  Bespreken, vinden zij dit ook bij gezond leven horen. Wat verstaan ze daaronder.  *Probing vragen:*   - Over welke onderwerpen over gezondheid weet je al wat dingen? - Over welke onderwerpen over gezondheid weet je niet zo veel? - Over welke onderwerpen over gezond leven wil je wel wat meer weten? - Wat wordt er thuis over gezond leven verteld, of gedaan? - Wat wordt er op school over gezond leven vertelt, of gedaan?   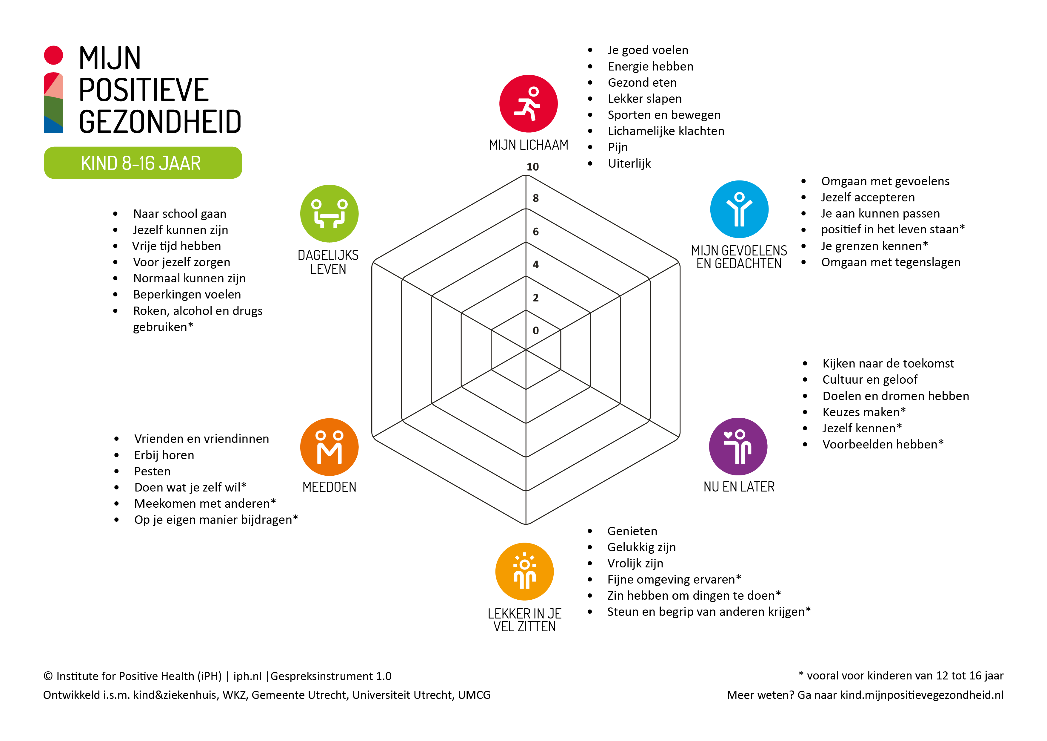  (Institute of Positive Health, n.d.) |
| Onderwerp 2:  Het vinden van informatie | Probing vragen:   - Op wat voor manier krijgen jullie informatie over gezondheid?   *Op school? Thuis? Online? Tv? Boek?*   - Als je iets wilt weten over gezond leven, vraag je dat dan aan iemand   *Zo ja, Aan wie, waarover? Krijg je dan antwoord?*   - Zoeken jullie zelf weleens naar informatie? - Waar doe je dat?   *Internet? Tv, bijvoorbeeld klokhuis? Boeken?*   - Lukt dat goed? Krijg je dan een antwoord op je vraag?   Extra vragen:  Hoe makkelijk of moeilijk is het om erachter te komen hoe je snel beter kunt worden als je verkouden bent  Hoe makkelijk of moeilijk is het om erachter te komen wat je kunt doen om niet te dik of te dun te worden?  Hoe makkelijk of moeilijk is het om erachter te komen hoe je het beste kunt ontspannen  Hoe makkelijk of moeilijk is het om erachter te komen welk eten voor jou gezond is? |
| Onderwerp 3:  Het begrijpen van de informatie | Probing vragen:   - Als je iets opzoekt over gezondheid, begrijp je het dan altijd? - Als school of je ouders je iets vertellen over gezondheid, begrijp je dat dan altijd? - Wat begrijp je wel, wat begrijp je niet? - Wat doe je als je het niet begrijpt?   *Vragen stellen, het zelf opzoeken?*  Extra vragen:  Hoe makkelijk of moeilijk is het om te begrijpen wanneer en hoe je je medicijnen moet innemen als je ziek bent?  Hoe makkelijk of moeilijk is het om te begrijpen waarom je soms naar de dokter moet, zelf als je helemaal niet ziek bent?  Hoe makkelijk of moeilijk is het om te begrijpen waarom je moet worden ingeënt (een prik krijgt)?  Hoe makkelijk of moeilijk is het om te begrijpen wat je ouders je vertellen over je gezondheid?  Hoe makkelijk of moeilijk is het om te begrijpen waarom je soms ook moet uitrusten |
| Onderwerp 4:  Het beoordelen van de informatie | Probing vragen:  *Als je dus informatie leest, of iemand vertelt je iets, of je hebt het zelf opgezocht...*   - Hoe weet je dan of de informatie waar is, of het klopt? - Waar let je op? - Is het van het ene betrouwbaarder dan van het andere? *Voorbeeld?* - Heb je wel eens iets gelezen, waarvan je dacht. Ik denk niet dat dit waar is? Wist je het toen zeker of twijfelde je? Waar was dit? - Wat doe je als je twijfelt? - Vinden jullie het soms lastig om te weten of iets waar is of niet?   Extra vragen:  Hoe makkelijk of moeilijk is het om te kiezen wat voor jou wel en niet helpt om gezond te blijven? |
| Onderwerp 5:  Het toepassen van de informatie | Probing vragen:   - Als iemand je iets vertelt over gezondheid, bijv. je ouders of op school, luister je hier dan naar en ga je dit dan ook doen? (Fruit eten, niet roken, 2x je tandenpoetsen, veel bewegen). - Doe je sommige dingen ook niet?   Omdat je het vergeet of omdat je het niet nodig vindt. (voorbeeld noemen.)   - Heb je wel eens iets geleerd of gelezen over gezondheid wat je nu ook doet. (wat dan?) - Heb je wel eens iets geleerd of gelezen over gezondheid wat je toch niet doet. (wat dan?) - Wanneer doe je iets dan wel, wanneer doe je iets niet. - Heb je soms nog hulp nodig om gezond te kunnen leven, bijv. je moeder die je helpt herinneren dat je fruit moeten eten, of je vader die zegt dat je je tanden moet poesten? Of lukt dit zelfstandig?   Voorbeeld vraag:   - Als je moeder zegt dat je elke dag fruit moet eten, maar je vriendjes doen dat niet. Wat doe jij dan?   Extra vragen:  Hoe makkelijk of moeilijk is het om te doen wat je ouders tegen je zeggen om weer beter te worden?  Hoe makkelijk of moeilijk is het om je medicijnen in te nemen zoals het je is verteld?  Hoe makkelijk of moeilijk is het om je te houden aan de verkeersregels die je hebt geleerd?  Hoe makkelijk of moeilijk is het om gezond te eten? |
| Stellingen | Tot slot heb ik nog stellingen die je mag beantwoorden met waar of niet waar.  Als je het er mee eens bent mag je je handopsteken.  (*Hardop tellen hoeveel waar en niet waar zijn, voor opname)*   - Ik voel me gezond - Ik vind het leuk om te leren over gezond leven. - Als ik iets wil weten over gezond leven, zoek ik zelf informatie op - Thuis zijn we veel bezig met gezondheid. - Op school zijn we veel bezig met gezondheid. - Ik heb hulp nodig om gezond te kunnen leven - Als de mensen om mij heen niet gezond leven, vind ik het lastig om wel gezond te leven. - Ik vind vrolijk zijn, belangrijker dan gezond eten. - Als ik gezond eet en sport, zit ik lekkerder in mijn vel. |
| Afsluiting: | Willen jullie zelf nog iets zeggen of toevoegen? Dingen die ik vergeten ben te vragen of die jij zelf belangrijk vindt?  Dan was dit het gesprek en wil ik jullie heel erg bedanken! |

## Adapted version – School C in Dutch

Hoofdvraag:

Wat vinden kinderen in de leeftijd 9-12 jaar belangrijk als het gaat om in het inzetten van gezondheidsvaardigheden om dagelijks gezond te kunnen leven?

Benodigdheden:

- Rustige ruimte
- Opname apparatuur (dictafoon)
- Voldoende A4 papier
- Pennen
- Namenlijst
- Dit protocol uitgeprint

Begin van elke opname melden hoeveel kinderen er aanwezig zijn bij het gesprek, welke klas en welke leeftijd.

| Welkom: |  |
| --- | --- |
| Regels: | - We luisteren naar elkaar en praten niet door elkaar heen - Je mag in gesprek gaan met elkaar - Er is geen antwoord goed of slecht. - Het is belangrijk dat je je eigen mening geeft, deze hoeft dus niet hetzelfde te zijn als die van je klasgenoot. - We lachen elkaar dus niet uit en hebben respect voor elkaars mening   Zijn er verder nog vragen? |
| Start focusgroep discussie | Voorstelrondje  Iedereen heeft een papier voor zich en een pen. Daar mag je even je leeftijd op schrijven, en het antwoord op de volgende vraag: |
| Onderwerp 1:  Gezondheids-onderwerpen | - Als het gaat over gezondheid, waar moeten jullie dan aan denken?   *Je mag het opschrijven, maar als je tekenen makkelijker vindt mag dat ook. Je mag meerdere dingen opschrijven en je mag er best even over nadenken.*  *Pennen inleveren, en antwoorden met elkaar doornemen. (Wanneer alleen gezond eten er uitkomt, uitleggen wat gezondheid nog meer inhoudt).*  *Probing vragen:*   - Over welke onderwerpen over gezondheid weet je al wat dingen? - Over welke onderwerpen over gezondheid weet je niet zo veel? - Over welke onderwerpen over gezondheid wil je wel wat meer weten? - Wat leer je thuis over gezondheid? - Wat leer je op school over gezondheid? |
| Onderwerp 2:  Het vinden van informatie | Probing vragen:   - Op wat voor manier krijgen jullie informatie over gezondheid?   *Op school? Thuis? Online? Tv? Boek?*   - Als je iets wilt weten over gezond leven, vraag je dat dan aan iemand   *Zo ja, Aan wie, waarover? Krijg je dan antwoord?*   - Zoeken jullie zelf weleens naar informatie? - Waar doe je dat?   *Internet? Tv, bijvoorbeeld klokhuis? Boeken?*   - Lukt dat goed? Krijg je dan een antwoord op je vraag?   **Vragenlijst begrijpelijkheid:**  **Hoe makkelijk of moeilijk is het om erachter te komen hoe je snel beter kunt worden als je verkouden bent**  **Hoe makkelijk of moeilijk is het om erachter te komen wat je kunt doen om niet te dik of te dun te worden?**  **Hoe makkelijk of moeilijk is het om erachter te komen hoe je het beste kunt ontspannen**  **Hoe makkelijk of moeilijk is het om erachter te komen welk eten voor jou gezond is?** |
| Onderwerp 3:  Het begrijpen van de informatie | Probing vragen:   - Als je iets opzoekt over gezondheid, begrijp je het dan altijd? - Als school of je ouders je iets vertellen over gezondheid, begrijp je dat dan altijd? - Wat begrijp je wel, wat begrijp je niet? - Wat doe je als je het niet begrijpt?   *Vragen stellen, het zelf opzoeken?*  **Vragenlijst begrijpelijkheid:**  **Hoe makkelijk of moeilijk is het om te begrijpen wanneer en hoe je je medicijnen moet innemen als je ziek bent?**  **Hoe makkelijk of moeilijk is het om te begrijpen waarom je soms naar de dokter moet, zelf als je helemaal niet ziek bent?**  **Hoe makkelijk of moeilijk is het om te begrijpen waarom je moet worden ingeënt (een prik krijgt)?**  **Hoe makkelijk of moeilijk is het om te begrijpen wat je ouders je vertellen over je gezondheid?**  **Hoe makkelijk of moeilijk is het om te begrijpen waarom je soms ook moet uitrusten** |
| Onderwerp 4:  Het beoordelen van de informatie | Probing vragen:  *Als je dus informatie leest, of iemand vertelt je iets, of je hebt het zelf opgezocht...*   - Hoe weet je dan of de informatie waar is, of het klopt? - Waar let je op? - Is het van het ene betrouwbaarder dan van het andere? *Voorbeeld?* - Heb je wel eens iets gelezen, waarvan je dacht. Ik denk niet dat dit waar is? Wist je het toen zeker of twijfelde je? Waar was dit? - Wat doe je als je twijfelt? - Vinden jullie het soms lastig om te weten of iets waar is of niet?   **Vragenlijst begrijpelijkheid:**  **Hoe makkelijk of moeilijk is het om te kiezen wat voor jou wel en niet helpt om gezond te blijven?** |
| Onderwerp 5:  Het toepassen van de informatie | Probing vragen:   - Als iemand je iets vertelt over gezondheid, bijv je ouders of op school, luister je hier dan naar en ga je dit dan ook doen? (Fruit eten, niet roken, 2x je tandenpoetsen, veel bewegen). - Doe je sommige dingen ook niet?   Omdat je het vergeet of omdat je het niet nodig vindt. (voorbeeld noemen.)   - Heb je wel eens iets geleerd of gelezen over gezondheid wat je nu ook doet. (wat dan?) - Heb je wel eens iets geleerd of gelezen over gezondheid wat je toch niet doet. (wat dan?) - Wanneer doe je iets dan wel, wanneer doe je iets niet. - Heb je soms nog hulp nodig om gezond te kunnen leven, bijv. je moeder die je helpt herinneren dat je fruit moeten eten, of je vader die zegt dat je je tanden moet poesten? Of lukt dit zelfstandig? - Als je moeder zegt dat je elke dag fruit moet eten, maar je vriendjes doen dat niet. Wat doe jij dan?   **Vragenlijst begrijpelijkheid:**  **Hoe makkelijk of moeilijk is het om te doen wat je ouders tegen je zeggen om weer beter te worden?**  **Hoe makkelijk of moeilijk is het om je medicijnen in te nemen zoals het je is verteld?**  **Hoe makkelijk of moeilijk is het om je te houden aan de verkeersregels die je hebt geleerd?**  **Hoe makkelijk of moeilijk is het om gezond te eten?** |
| Meest belangrijke onderwerpen van gezondheid | Als je naar het spinnenweb (mijn positieve gezondheid, kindtool) kijkt, omcirkel dan de meest belangrijke onderwerpen voor jou als het gaat om gezondheid. |
| Afsluiting: | Willen jullie zelf nog iets zeggen of toevoegen? Dingen die ik vergeten ben te vragen of die jij zelf belangrijk vindt?  Dan was dit het gesprek en wil ik jullie heel erg bedanken! |

## COREQ (COnsolidated criteria for REporting Qualitative research) Checklist

A checklist of items that should be included in reports of qualitative research. You must report the page number in your manuscript where you consider each of the items listed in this checklist. If you have not included this information, either revise your manuscript accordingly before submitting or note N/A.

| **Topic** | **Item No.** | **Guide Questions/Description** | **Reported on**  **Page No.** |
| --- | --- | --- | --- |
| **Domain 1: Research team**  **and reﬂexivity** | | | |
| *Personal characteristics* | | | |
| Interviewer/facilitator | 1 | Which author/s conducted the interview or focus group? | 5-6 |
| Credentials | 2 | What were the researcher’s credentials? E.g. PhD, MD | 1 |
| Occupation | 3 | What was their occupation at the time of the study? | 1 |
| Gender | 4 | Was the researcher male or female? | 1 |
| Experience and training | 5 | What experience or training did the researcher have? | 1 |
| *Relationship with*  *participants* | | | |
| Relationship established | 6 | Was a relationship established prior to study commencement? | No |
| Participant knowledge of  the interviewer | 7 | What did the participants know about the researcher? e.g. personal  goals, reasons for doing the research |  |
|  |  |  | Protocol |
|  |  |  |  |
| Interviewer characteristics | 8 | What characteristics were reported about the inter viewer/facilitator?  e.g. Bias, assumptions, reasons and interests in the research topic |  |
|  |  |  | Protocol |
|  |  |  |  |
| **Domain 2: Study design** | | | |
| *Theoretical framework* | | | |
| Methodological orientation and Theory | 9 | What methodological orientation was stated to underpin the study? e.g. grounded theory, discourse analysis, ethnography, phenomenology,  content analysis |  |
|  |  |  | NA (explorativ |
|  |  |  |  |
| *Participant selection* | | | |
| Sampling | 10 | How were participants selected? e.g. purposive, convenience,  consecutive, snowball |  |
|  |  |  | 5 |
|  |  |  |  |
| Method of approach | 11 | How were participants approached? e.g. face-to-face, telephone, mail,  email |  |
|  |  |  | 5 |
|  |  |  |  |
| Sample size | 12 | How many participants were in the study? | 7 |
| Non-participation | 13 | How many people refused to participate or dropped out? Reasons? | 7 |
| *Setting* | | | |
| Setting of data collection | 14 | Where was the data collected? e.g. home, clinic, workplace | 5-6 |
| Presence of non-  participants | 15 | Was anyone else present besides the participants and researchers? |  |
|  |  |  | 5-6 |
|  |  |  |  |
| Description of sample | 16 | What are the important characteristics of the sample? e.g. demographic  data, date |  |
|  |  |  | 7 |
|  |  |  |  |
| *Data collection* | | | |
| Interview guide | 17 | Were questions, prompts, guides provided by the authors? Was it pilot  tested? | protocol suppl |
|  |  |  |  |
| Repeat interviews | 18 | Were repeat inter views carried out? If yes, how many? | NA |
| Audio/visual recording | 19 | Did the research use audio or visual recording to collect the data? | 6 |
| Field notes | 20 | Were ﬁeld notes made during and/or after the inter view or focus group? | NA |
| Duration | 21 | What was the duration of the inter views or focus group? | 6 |
| Data saturation | 22 | Was data saturation discussed? | NA |
| Transcripts returned | 23 | Were transcripts returned to participants for comment and/or correction? | NA |
| **Domain 3: analysis and**  **ﬁndings** | | | |
| *Data analysis* | | | |
| Number of data coders | 24 | How many data coders coded the data? | 6 |
| Description of the coding  tree | 25 | Did authors provide a description of the coding tree? |  |
|  |  |  | 8 and suppl 1 |
|  |  |  |  |
| Derivation of themes | 26 | Were themes identiﬁed in advance or derived from the data? | 6-8 |
| Software | 27 | What software, if applicable, was used to manage the data? | 6 |
| Participant checking | 28 | Did participants provide feedback on the ﬁndings? | NA |
| *Reporting* | | | |
| Quotations presented | 29 | Were participant quotations presented to illustrate the themes/ﬁndings?  Was each quotation identiﬁed? e.g. participant number |  |
|  |  |  | 9-16 |
|  |  |  |  |
| Data and ﬁndings consistent | 30 | Was there consistency between the data presented and the ﬁndings? | 9-16 |
| Clarity of major themes | 31 | Were major themes clearly presented in the ﬁndings? | 8-16 |
| Clarity of minor themes | 32 | Is there a description of diverse cases or discussion of minor themes? | 9-16 |
|  |  |  |  |

### Code tree

| **Category from protocol** | **Code** | **Sub theme** | **Theme English** |
| --- | --- | --- | --- |
| ONDERWERPEN denken aan | fit zijn | bodily functions | Health and healthy living |
| ONDERWERPEN denken aan | Hygiene | bodily functions | Health and healthy living |
| ONDERWERPEN denken aan | Slapen | bodily functions | Health and healthy living |
| KENNIS geen | Dagelijks leven | Daily Life | Health and healthy living |
| KENNIS wel | dagelijksleven | Daily Life | Health and healthy living |
| KENNIS wel | Energie hebben | Daily Life | Health and healthy living |
| TOEPASSEN niet | geen zin | Daily Life | Health and healthy living |
| TOEPASSEN niet | Kan niets bedenken | Daily Life | Health and healthy living |
| TOEPASSEN niet | Levert niks op | Daily Life | Health and healthy living |
| TOEPASSEN niet | niet toepassen | Daily Life | Health and healthy living |
| TOEPASSEN niet | Vergeten | Daily Life | Health and healthy living |
| TOEPASSEN wel | Afspraken | Daily Life | Health and healthy living |
| TOEPASSEN wel | bij beloning | Daily Life | Health and healthy living |
| TOEPASSEN wel | wel toepassen | Daily Life | Health and healthy living |
| TOEPASSEN wel | Zelf beslissen | Daily Life | Health and healthy living |
| ONDERWERPEN belangrijk | voor jezelf zorgen | Daily Life | Health and healthy living |
| ONDERWERPEN belangrijk | vrije tijd | Daily Life | Health and healthy living |
| ONDERWERPEN denken aan | gamen | Daily Life | Health and healthy living |
| ONDERWERPEN denken aan | geen middelen gebruik | Daily Life | Health and healthy living |
| ONDERWERPEN denken aan | Ontspannen | Daily Life | Health and healthy living |
| ONDERWERPEN denken aan | School | Daily Life | Health and healthy living |
| ONDERWERPEN denken aan | werken | Daily Life | Health and healthy living |
| ONDERWERPEN denken aan | Buiten zijn | Daily Life | Health and healthy living |
| ONDERWERPEN denken aan | Omgeving | Daily Life | Health and healthy living |
| KENNIS geen | Gezond eten/drinken | Diet | Health and healthy living |
| KENNIS wel | Gezond eten | Diet | Health and healthy living |
| ONDERWERPEN denken aan | Gezond eten | Diet | Health and healthy living |
| ONDERWERPEN belangrijk | Vrolijk zijn | Feelings and thoughts | Health and healthy living |
| ONDERWERPEN denken aan | Emoties | Feelings and thoughts | Health and healthy living |
| ONDERWERPEN denken aan | Praten, | Feelings and thoughts | Health and healthy living |
| ONDERWERPEN belangrijk | Zin hebben om dingen te doen | Feelings and thoughts | Health and healthy living |
| KENNIS geen | Ziekenhuis | healthcare | Health and healthy living |
| KENNIS geen | Ziekte | healthcare | Health and healthy living |
| KENNIS wel | beperkingen voelen | healthcare | Health and healthy living |
| KENNIS wel | Ziekte | healthcare | Health and healthy living |
| KENNIS wel | Zorgprofessional/Zorg locatie | healthcare | Health and healthy living |
| TOEPASSEN wel | Medicijnen | healthcare | Health and healthy living |
| TOEPASSEN wel | Ongezond voelen | healthcare | Health and healthy living |
| ONDERWERPEN denken aan | Medicijnen | healthcare | Health and healthy living |
| ONDERWERPEN denken aan | Ziekte | healthcare | Health and healthy living |
| ONDERWERPEN belangrijk | voorbeelden hebben | now and in the future | Health and healthy living |
| ONDERWERPEN denken aan | Eigen keuzes maken | now and in the future | Health and healthy living |
| ONDERWERPEN belangrijk | vrienden | Participation | Health and healthy living |
| ONDERWERPEN denken aan | goed behandeld worden | Participation | Health and healthy living |
| TOEPASSEN niet | invloed omgeving | participation | Health and healthy living |
| KENNIS wel | Bewegen | Physical Activity | Health and healthy living |
| TOEPASSEN wel | Bewegen | Physical Activity | Health and healthy living |
| ONDERWERPEN denken aan | Bewegen | Physical Activity | Health and healthy living |
| ONDERWERPEN denken aan | Sport | Physical Activity | Health and healthy living |
| KENNIS geen | Wat is gezond | Health topics | Information for healthy living |
| LEREN/KRIJGEN informatie school | Bewegen | Health topics | Information for healthy living |
| LEREN/KRIJGEN informatie school | Gezond eten | Health topics | Information for healthy living |
| LEREN/KRIJGEN informatie school | Meer buiten | Health topics | Information for healthy living |
| LEREN/KRIJGEN informatie school | met elkaar omgaan | Health topics | Information for healthy living |
| LEREN/KRIJGEN informatie thuis | Bewegen | Health topics | Information for healthy living |
| LEREN/KRIJGEN informatie thuis | gezond eten | Health topics | Information for healthy living |
| LEREN/KRIJGEN informatie thuis | hygiene | Health topics | Information for healthy living |
| LEREN/KRIJGEN informatie thuis | Roken alcohol | Health topics | Information for healthy living |
| LEREN/KRIJGEN informatie thuis | Slaap | Health topics | Information for healthy living |
| ZOEKEN/VINDEN wel | wat gezond is | Health topics | Information for healthy living |
| LEREN/KRIJGEN informatie thuis |  | Receiving information in daily life | Information for healthy living |
| LEREN/KRIJGEN informatie thuis | Geen gezondheidsinformatie | Receiving information in daily life | Information for healthy living |
| LEREN/KRIJGEN informatie thuis | Wel gezondheidsinformatie | Receiving information in daily life | Information for healthy living |
| LEREN/KRIJGEN informatie school | School gezondheid | Receiving information in daily life | Information for healthy living |
| LEREN/KRIJGEN informatie school | Weinig specifiek over gezondheid | Receiving information in daily life | Information for healthy living |
| BEGRIJPEN niet | Doorvragen ouders/juf | Seeking help | Information for healthy living |
| TOEPASSEN wel | Geen hulp nodig | Seeking help | Information for healthy living |
| TOEPASSEN wel | Hulp nodig | Seeking help | Information for healthy living |
| BEGRIJPEN niet | doen alsof wel begrijpen | Seeking help | Information for healthy living |
| BEGRIJPEN niet | geen begrip maar wel uitleg | Seeking help | Information for healthy living |
| KENNIS willen hebben | slaap | Learning about health | Health and healthy living |
| KENNIS willen hebben | Lichaam | Learning about health | Health and healthy living |
| KENNIS willen hebben | Dagelijks leven | Learning about health | Health and healthy living |
| KENNIS willen hebben | calorieen/voedingswaarde product | Learning about health | Health and healthy living |
| KENNIS willen hebben | Toekomst | Learning about health | Health and healthy living |
| KENNIS willen hebben | Ziekte | Learning about health | Health and healthy living |
| KENNIS willen hebben | leren gezondheid | Learning about health | Health and healthy living |
| KENNIS willen hebben | Geen informatie willen | Learning about health | Health and healthy living |
| KENNIS willen hebben | Niet weten welk onderwerp | Learning about health | Health and healthy living |
| KENNIS willen hebben | Sport/bewegen | Physical activity | Health and healthy living |
| BEGRIJPEN niet | moeilijke woorden | Language and accessibility of information | Information for healthy living |
| BEGRIJPEN niet | niet begrijpen algemeen | Language and accessibility of information | Information for healthy living |
| BEGRIJPEN niet | Niet begrijpen opgeven | Language and accessibility of information | Information for healthy living |
| BEGRIJPEN niet | Niet goed luisteren | Language and accessibility of information | Information for healthy living |
| BEGRIJPEN niet | Snel praten | Language and accessibility of information | Information for healthy living |
| BEGRIJPEN niet | Te uitgebreid | Language and accessibility of information | Information for healthy living |
| BEGRIJPEN wel | Dikgedrukte woorden | Language and accessibility of information | Information for healthy living |
| BEGRIJPEN wel | Juiste zoekopdracht invoeren | Language and accessibility of information | Information for healthy living |
| BEGRIJPEN wel | kort antwoord | Language and accessibility of information | Information for healthy living |
| BEGRIJPEN wel | Makkelijke taal | Language and accessibility of information | Information for healthy living |
| BEGRIJPEN wel | antwoord op vraag | Language and accessibility of information | Information for healthy living |
| ZOEKEN/VINDEN wel | opzoeken informatie | Media/online information | Sources of information for healthy living |
| BEGRIJPEN niet | Doorzoeken online | Media/online information | Sources of information for healthy living |
| ZOEKEN/VINDEN wel | Google | Media/online information | Sources of information for healthy living |
| ZOEKEN/VINDEN wel | Snapchat | Media/online information | Sources of information for healthy living |
| ZOEKEN/VINDEN wel | TV | Media/online information | Sources of information for healthy living |
| ZOEKEN/VINDEN wel | Wiki pedia | Media/online information | Sources of information for healthy living |
| ZOEKEN/VINDEN wel | Youtube en tiktok | Media/online information | Sources of information for healthy living |
| BRONNEN gezondheidsinformatie | Filmpje | Media/online information | Sources of information for healthy living |
| BRONNEN gezondheidsinformatie | Google | Media/online information | Sources of information for healthy living |
| BRONNEN gezondheidsinformatie | programma | Media/online information | Sources of information for healthy living |
| BRONNEN gezondheidsinformatie | Tiktok | Media/online information | Sources of information for healthy living |
| BRONNEN gezondheidsinformatie | Wiki pedia | Media/online information | Sources of information for healthy living |
| BRONNEN gezondheidsinformatie | Youtube | Media/online information | Sources of information for healthy living |
| ZOEKEN/VINDEN wel | School | professionals | Sources of information for healthy living |
| ZOEKEN/VINDEN wel | vragen aan dokter | professionals | Sources of information for healthy living |
| BRONNEN gezondheidsinformatie | dokter | professionals | Sources of information for healthy living |
| BRONNEN gezondheidsinformatie | School/Docent | professionals | Sources of information for healthy living |
| TOEPASSEN niet | Advies tandarts | professionals | Sources of information for healthy living |
| ZOEKEN/VINDEN wel | Op sport | Social contacts | Sources of information for healthy living |
| ZOEKEN/VINDEN wel | vragen aan ouders/familie | Social contacts | Sources of information for healthy living |
| BRONNEN gezondheidsinformatie | Ouders | Social contacts | Sources of information for healthy living |
| BRONNEN gezondheidsinformatie | Zelf ontdekt | Social contacts | Sources of information for healthy living |
| BEOORDELEN betrouwbaarheid | Vragen ouders | Social contacts | Sources of information for healthy living |
| TOEPASSEN niet | advies ouders | Social contacts | Sources of information for healthy living |
| TOEPASSEN wel | Advies geven aan ander | Social contacts | Sources of information for healthy living |
| TOEPASSEN wel | Advies ouders | Social contacts | Sources of information for healthy living |
| BEGRIJPEN niet | Ouders weten ook niet alles | Trustworthiness of sources | Sources of information for healthy living |
| BEOORDELEN betrouwbaarheid | Beoordelen moeilijk | Trustworthiness of sources | Sources of information for healthy living |
| BEOORDELEN betrouwbaarheid | betrouwbare bron | Trustworthiness of sources | Sources of information for healthy living |
| BEOORDELEN betrouwbaarheid | Bovenste zoekresultaat betrouwbaar | Trustworthiness of sources | Sources of information for healthy living |
| BEOORDELEN betrouwbaarheid | Ge-edit | Trustworthiness of sources | Sources of information for healthy living |
| BEOORDELEN betrouwbaarheid | meerderheid zegt hetzelfde | Trustworthiness of sources | Sources of information for healthy living |
| BEOORDELEN betrouwbaarheid | Nep info/reclames | Trustworthiness of sources | Sources of information for healthy living |
| BEOORDELEN betrouwbaarheid | vertrouwen internet | Trustworthiness of sources | Sources of information for healthy living |
| BEOORDELEN betrouwbaarheid | Waarheid | Trustworthiness of sources | Sources of information for healthy living |
| BEOORDELEN bruikbaarheid | Vergelijken andere bronnen | Trustworthiness of sources | Sources of information for healthy living |
| TOEPASSEN niet | Bron van info | Trustworthiness of sources | Sources of information for healthy living |
| TOEPASSEN wel | Bron is betrouwbaar | Trustworthiness of sources | Sources of information for healthy living |
| BEGRIJPEN niet | Geen nuttige informatie | Usefullness of sources | Sources of information for healthy living |
| BEOORDELEN bruikbaarheid | Antwoord op de vraag | Usefullness of sources | Sources of information for healthy living |
| BEOORDELEN bruikbaarheid | Beste zoekresultaat | Usefullness of sources | Sources of information for healthy living |
| BEOORDELEN bruikbaarheid | Duidelijkheid in wat gezond is | Usefullness of sources | Sources of information for healthy living |
| BEOORDELEN bruikbaarheid | Juiste informatiebron gebruiken | Usefullness of sources | Sources of information for healthy living |
| ZOEKEN/VINDEN wel | Boek | written information offline | Sources of information for healthy living |
| BRONNEN gezondheidsinformatie | boek | written information offline | Sources of information for healthy living |
| BRONNEN gezondheidsinformatie | Verpakking product | written information offline | Sources of information for healthy living |

### Code networks for themes from Atlas.ti in Dutch

Theme 1: Perspectives on Health and Health literacy


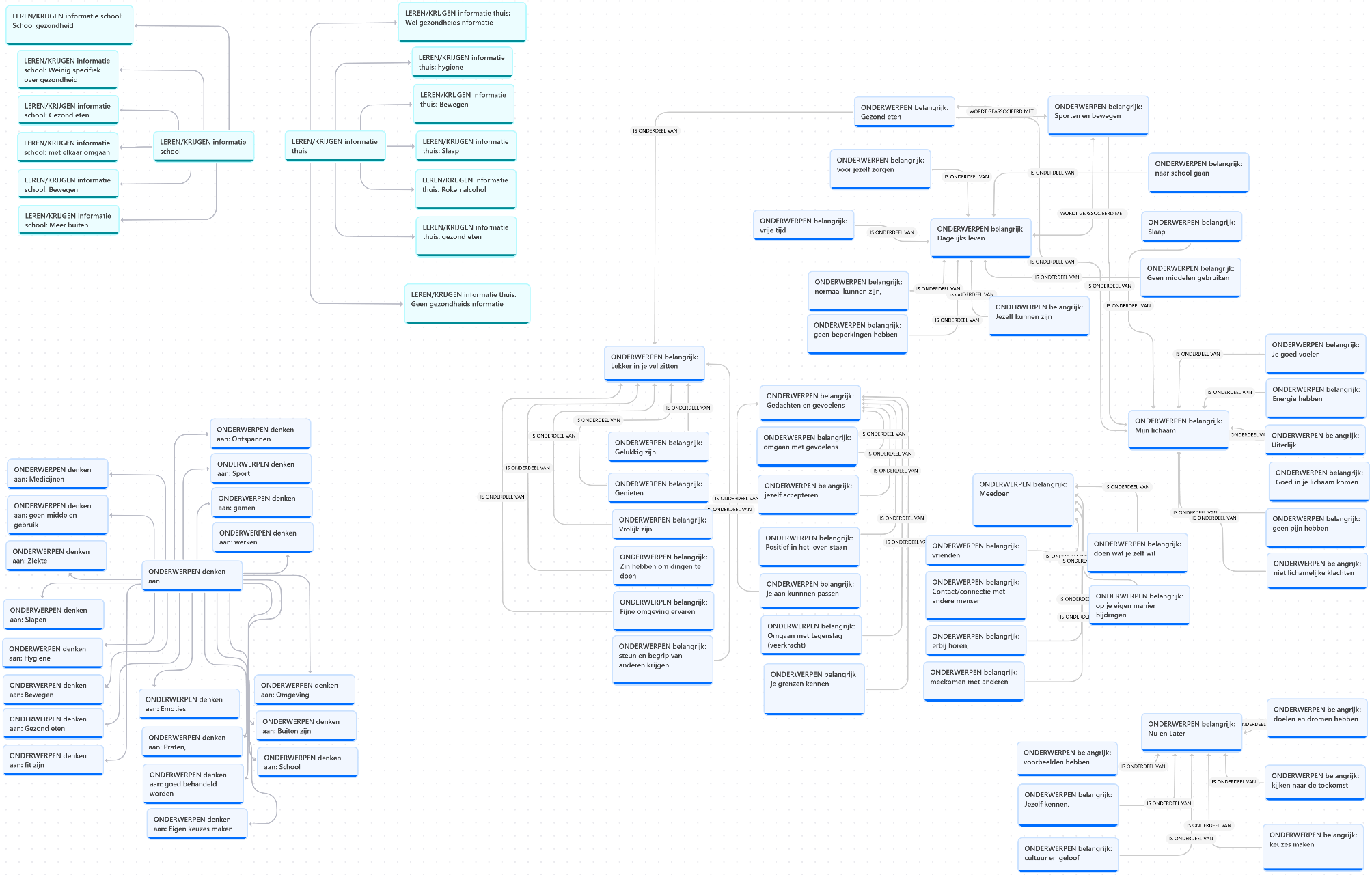


Theme 2: Information for healthy living


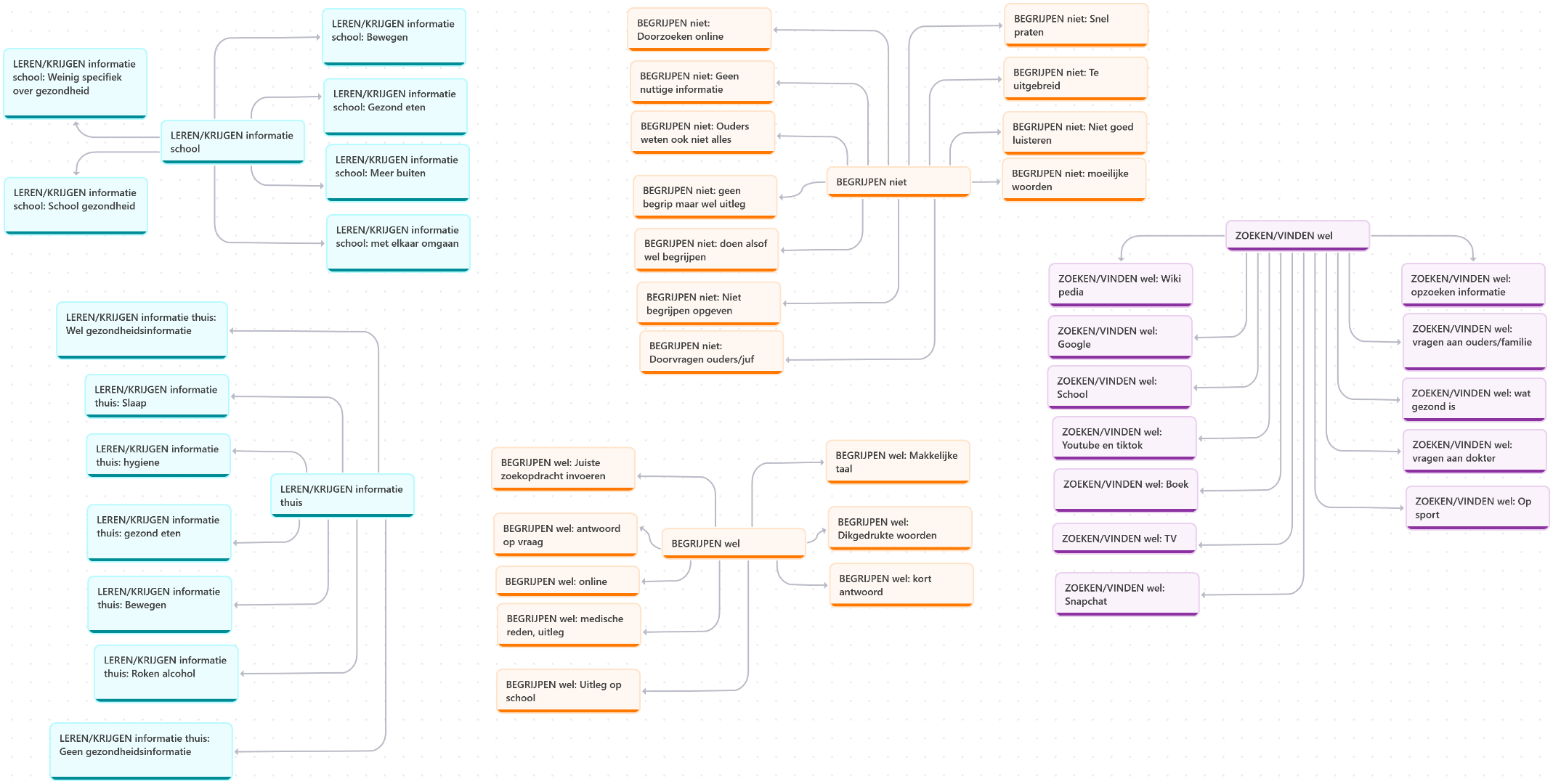


Theme 3: Sources of information for healthy living


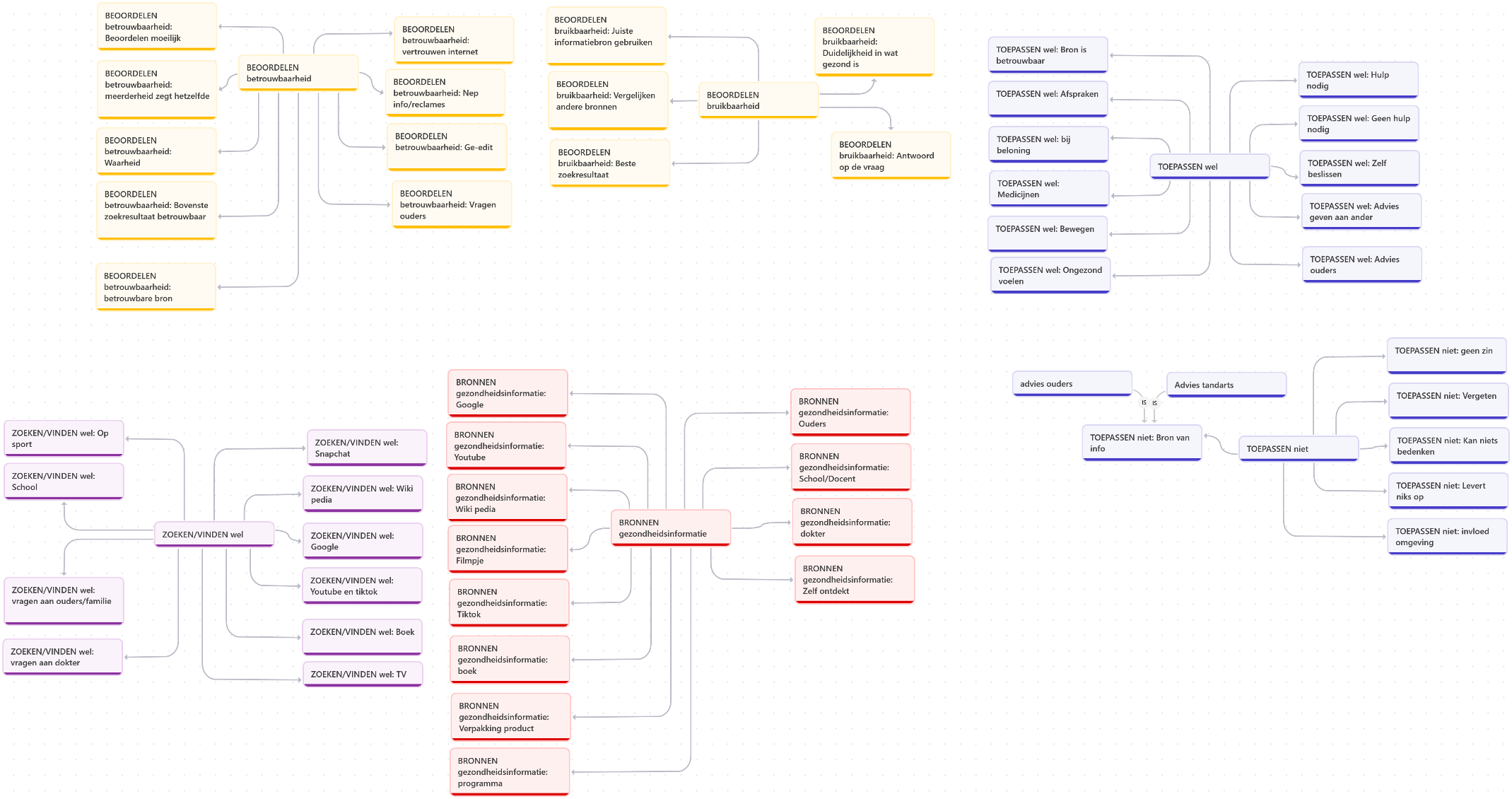


### Code networks of HL aspects

Searching


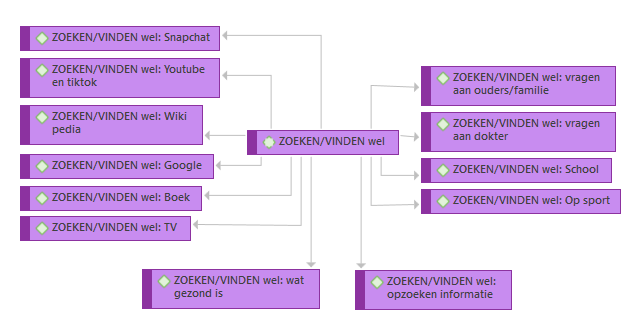


Understanding


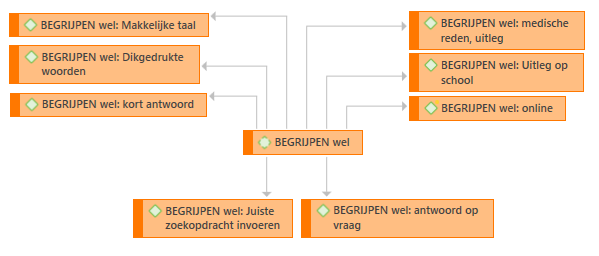


Not understanding


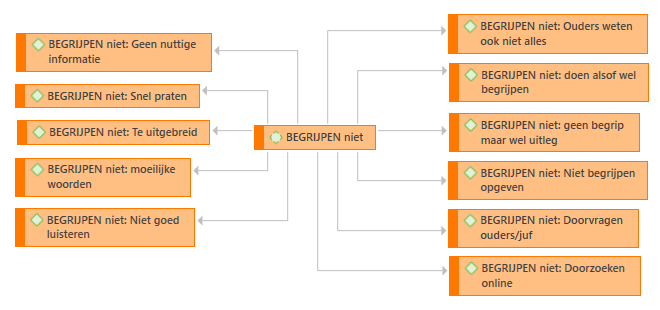


Appraising
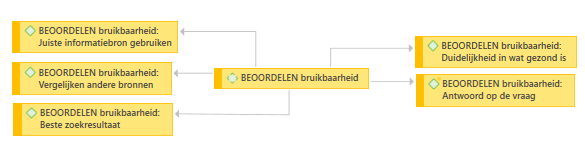
– usefulness

Appraising – Trustworthiness
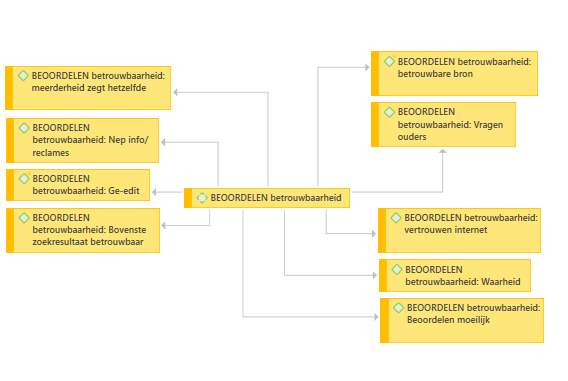


Applying **
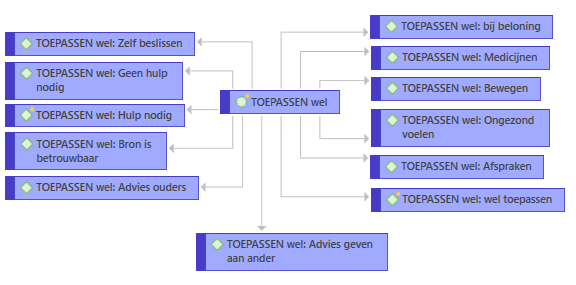
**

Applying - not **
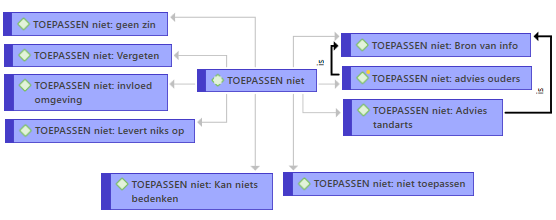
**

**Knowledge**


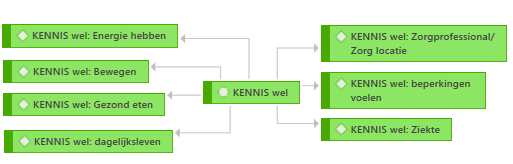


Knowledge


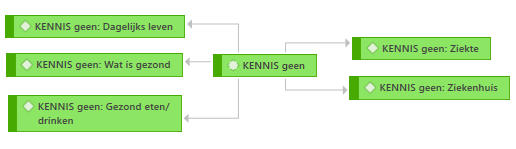


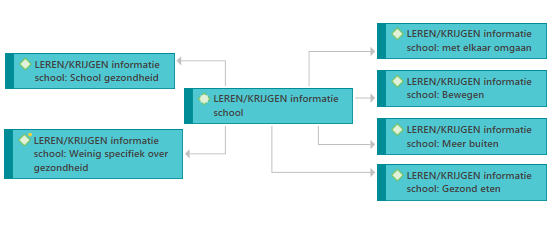
Learning about information – School

Learning about information – at home

**
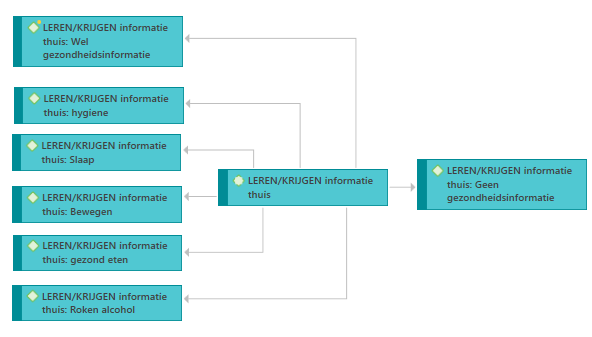
**

Sources of information


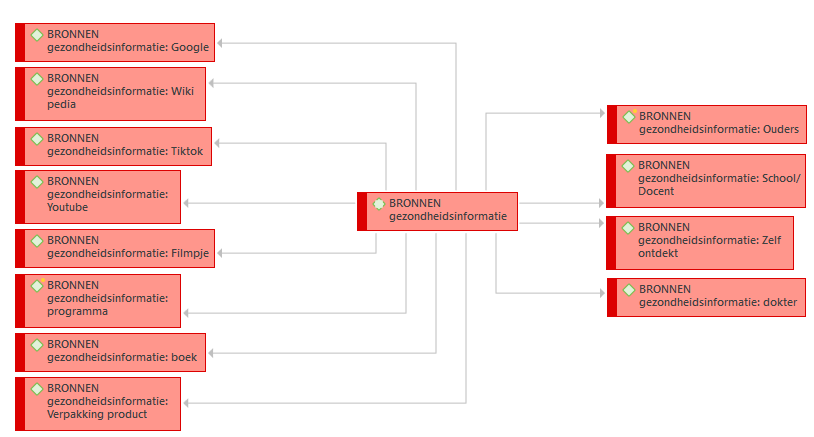

Supplement: daae208_suppl_Supplementary_Files [file daae208_suppl_supplementary_files.docx]
